# Does free tendon length influence the injury risk of the Achilles tendon? A finite element study

**DOI:** 10.1002/jeo2.70036

**Published:** 2024-11-14

**Authors:** Pedro Diniz, Carlos Quental, Hélder Pereira, André Soares Ferreira, Gino M. M. J. Kerkhoffs, Frederico Castelo Ferreira, João Folgado

**Affiliations:** ^1^ Department of Orthopaedic Surgery Centre Hospitalier de Luxembourg – Clinique d'Eich Luxembourg Luxembourg; ^2^ Department of Bioengineering and iBB Institute for Bioengineering and Biosciences, Instituto Superior Técnico, Universidade de Lisboa Lisbon Portugal; ^3^ Associate Laboratory i4HB – Institute for Health and Bioeconomy, Instituto Superior Técnico Universidade de Lisboa Lisbon Portugal; ^4^ Luxembourg Institute of Research in Orthopedics, Sports Medicine and Science (LIROMS) Luxembourg Luxembourg; ^5^ Luxembourg Institute of Health (LIH) Luxembourg Luxembourg; ^6^ IDMEC, Instituto Superior Técnico Universidade de Lisboa Lisbon Portugal; ^7^ Department of Orthopaedic Centro Hospitalar Póvoa de Varzim Vila do Conde Portugal; ^8^ Ripoll y De Prado Sports Clinic: FIFA Medical Centre of Excellence, Murcia‐Madrid Murcia Spain; ^9^ University of Minho ICVS/3B's – PT Government Associate Laboratory Braga/Guimarães Portugal; ^10^ Department of Orthopaedic Surgery Hospital de Sant'Ana Parede Portugal; ^11^ Department of Orthopaedic Surgery, Amsterdam Movement Sciences Amsterdam University Medical Centers; Academic Center for Evidence Based Sports Medicine (ACES); Amsterdam Collaboration for Health and Safety in Sports (ACHSS) Amsterdam The Netherlands

**Keywords:** Achilles tendon, finite element modelling, injury risk factors, tendon rupture

## Abstract

**Purpose:**

The Achilles tendon is a common injury site, but anatomical risk factors for injury are relatively unexplored in the literature. This study aimed to evaluate whether changes in free tendon length would influence the results of a simulated rupture of the Achilles tendon.

**Methods:**

Using a previously validated 3D finite element model of the free and aponeurotic Achilles tendon as a basis, two additional finite element models with 25% decreased and increased free tendon lengths were created. The finite element models were sequentially loaded from 2500 to 3500N in 100N increments, and the total volume of elements exhibiting a maximal principal strain above 10% was recorded. An Achilles tendon rupture was considered to have occurred when a continuous group of elements with a volume of at least 3 mm^3^ exhibited a maximum principal strain above 10%. Models were compared regarding the smallest load that met the rupture criterion and plots of the percentage of elements exhibiting maximum principal strains above 10% across the loading range. Sensitivity analyses assessed the influence of subtendon division variations and subtendon sliding restriction on the results.

**Results:**

Rupture loads and plots of the percentage of elements with maximum principal strains above 10% were similar between models, regardless of the free tendon length. No models met the rupture criterion when simulations were run without subtendon sliding. Rupture loads in the subtendon division variation models were correlated with the subtendon cross‐sectional areas.

**Conclusions:**

The simulated rupture results of the Achilles tendon were sensitive to variations in subtendon cross‐sectional areas but not in free tendon length.

**Level of Evidence:**

Level V.

**Abbreviations**
AT: Achilles tendon
CI: confidence interval
CSA: cross‐sectional area
FE: finite element
FFD: free‐form deformation
MPS: maximum principal strain
MRI: magnetic resonance imaging
MTJ: myotendinous junction

## INTRODUCTION

The Achilles tendon (AT) is a common site of injury. Disorders of AT can be divided into ruptures and overuse injuries [23]. The latter include midportion and insertional tendinopathy, paratendinopathy and superficial and retrocalcaneal bursitis [13]. Midportion tendinopathy is the most common of the overuse injuries [23]. The prevalence of midportion AT tendinopathy in the general adult population is 0.24% [24]. Still, it can be significantly higher in active populations, such as recreative runners, where it has been reported to be 5.2% in a cohort of 1929 subjects [32]. Studies evaluating the occurrence of AT rupture in patients with previously diagnosed midportion tendinopathy have reported a prevalence of 4.0%–14.6% [36, 38, 59], with the incidence of AT ruptures in the general population being reported to be 11.2–30.87 per 100,000 person‐years and seems to be increasing [18, 33, 40, 46], possibly owing to increased participation of older adults in sports [34].

The underlying cause of this disorder has been suggested to be the result of an imbalance between ‘protective/regenerative responses and damaging/degenerative changes’ that may be the outcome of chronic tendon overloading, metabolic diseases [2], or vascular impairments [7]. When those changes are extensive enough or large loads are involved, rupture may occur [11]. Accordingly, degenerative changes are expected in histological examinations of patients with AT ruptures [27, 37, 56].

The aetiology of tendinopathy is multifactorial [23, 51], with extrinsic and intrinsic factors being implicated. Extrinsic factors include general factors, such as the use of fluoroquinolones or corticosteroids [30] and sports‐related factors, which include overtraining and environmental conditions (e.g., hot or cold weather) [22, 26]. Intrinsic factors include systemic factors, such as gender, age and metabolic disorders (e.g., obesity and hypercholesterolaemia) [1, 51] and local factors, such as lower limb biomechanics [6] and AT structural anatomy [51].

The influence of AT geometry in its mechanical behaviour has been the focus of previous finite element (FE) studies. A study by Hansen et al. [21] has shown that stress distribution in the free tendon is more sensitive to subject‐specific geometry than material properties, with similar findings reported in other studies [47, 49, 60]. Another study by Shim et al. [48], aimed at assessing how the subtendon twist affected stress distribution and rupture load in the AT, reported that a twist angle of 30 degrees led to an improvement in AT strength of up to 40%. From these studies, it can be garnered that some aspects of the AT geometry may influence injury risk profiles. However, an important limitation of these studies is that they were focused only on the free tendon. In contrast, recent research has shown the possible involvement of aponeurotic AT in tendinopathy and rupture cases [43]. Despite its clinical relevance, an improved understanding of how specific geometrical features may impact the mechanical behaviour of the AT and its risk of injury still needs to be added to the current literature.

The objective of this study was to evaluate how changes in free tendon length influence the injury risk of the AT using a previously validated 3D FE model of the aponeurotic and free AT. It was hypothesized that an increased free tendon length could be a risk factor for AT injury.

The primary objective of this study was to investigate the impact of variations in free tendon length on the injury risk of the AT, using a previously validated three‐dimensional FE model that includes both the aponeurotic and free portions of the AT. It was hypothesized that an increased free tendon length might elevate the risk of injury in the form of increased strain during simulated loading of the AT. Our rationale stems from the biomechanical principle that tendon geometry can significantly influence stress distribution and mechanical behaviour, as evidenced by previous studies [21]. By systematically varying the free tendon length in our FE model, we aimed to identify whether these geometric changes could independently alter the strain patterns and rupture loads, thus contributing to the understanding of potential risk factors for AT injuries.

## MATERIALS AND METHODS

For the development of AT models with different free tendon lengths, this study uses a previously validated FE model of the AT [15], which is briefly described in the following sections. For a more in‐depth description of this model and its validation, the interested reader can referred to Ref. [15].

### Finite element models

#### Geometrical models

A 3D model of the free and aponeurotic AT was created, using a previously described software pipeline [45], from magnetic resonance imaging (MRIs) scans of a 34‐year‐old male (height: 180 cm; mass: 77 Kg), healthy and recreationally active, with no previous injury to the AT. An ultrasound scan was performed to estimate the total soleus muscle volume based on the formula described by Bandholm et al. [5].

**Figure 1:**
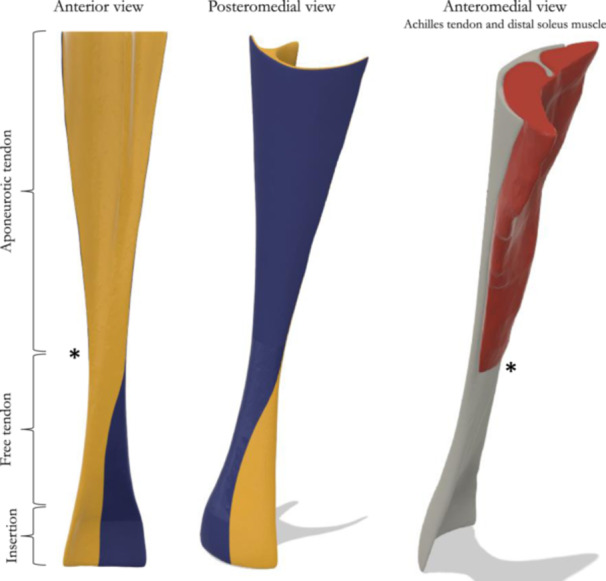
General overview of the baseline model. *: myotendinous junction.

In this model, hereafter referenced as the ‘baseline model’, a moderate degree of twisting was implemented, as described by Edama et al. [16], between the gastrocnemius and soleus subtendons. The distal soleus muscle was added to the model to estimate muscle volume and fibre directions (Figure 1).

The baseline model was then imported into Blender [10], where a free‐form deformation (FFD) algorithm was used to create geometrical variation models, as performed in previous FE studies [21, 47, 48, 49]. A lattice with 12 vertices distributed across three levels was made, centred on the distal myotendinous junction (MTJ) of the soleus muscle and aligned with the model's longitudinal and transversal axes. The four midway points were moved in the longitudinal direction, at a distance corresponding to 25% of the free tendon length, to generate free tendon length variation models while minimizing changes to other geometric features. Two AT models, with 25% larger and smaller free tendon lengths with respect to the baseline model, were generated.

The resulting AT and soleus muscle models were imported into Abaqus 6.14 (Dassault Systèmes) to generate 3D FE models. The AT and soleus muscle structures were meshed using hybrid quadratic tetrahedral elements (C3D10H).

#### Material model

The AT was represented as a transversely isotropic hyperelastic material. This study uses the constitutive model proposed by Gasser et al. (2006) [19], in which the strain energy function $\bar{\Psi }$ for an incompressible material is represented as

(1) $$\bar{\Psi }=\left\{\begin{array}{c} C_{10}\left({\overset{®}{I}}_{1}-3\right)\\ C_{10}\left({\overset{®}{I}}_{1}-3\right)+\frac{k_{1}}{{2k}_{2}}\left(e^{{k_{2}(k\left({\overset{®}{I}}_{1}-3\right)+(1-3k)({\overset{®}{I}}_{4}-1))}^{2}}-1\right) \end{array},\;\begin{array}{c} {\overset{®}{I}}_{4}<1\\ {\overset{®}{I}}_{4}\geq 1 \end{array}\right.,$$

where *C*_*10*_, *k*_*1*_, *k*_*2*_ and *k* are material parameters, *I*_1_ is the first strain invariant and *I*_4_ is the fourth strain invariant. Tendon fibre directions were estimated using a heat flux simulation on each subtendon [8]. Excluding the parameter *k*, the constitutive parameters were obtained by fitting the material model to experimental data [47]. Since no specific data on the AT was found for the parameter *k*, data on the supraspinatus tendon was used [25]. The material properties used in the FE analyses are summarized in Table 1.

**Table 1:** Material parameters used in the finite element analyses.

Material parameters	
C10	30.549
D	0
k1	327.345
k2	0.01
*k* [25]	0.139

#### Loading, boundary and interaction conditions

The loading conditions simulated an isometric plantarflexion contraction with the ankle in a neutral position. Loads were distributed by the soleus, medial and lateral gastrocnemius in a 62%, 26% and 12% proportion, respectively [3]. The distal soleus muscle, pulling on the ventral surface of the aponeurotic portion of the AT, was estimated to produce 6.52% of the total soleus muscle load [29]. The AT insertion was fixed in the x, y and z directions. In addition, the proximal surfaces of the tendon were constrained to move only in the direction of the force loads applied.

The subtendons interface was modelled using an anisotropic contact formulation without separation, for which friction coefficients of 1 and 0 were defined for the transverse and longitudinal directions, respectively. Such contact formulation was used to allow subtendon sliding in the longitudinal direction, a feature previously deemed necessary for accurate simulation of subtendon dynamics [20] and provide some degree of restriction against transversal plane sliding, based on the assumption that transverse sliding may be limited in vivo by the subtendon interface and the presence of the paratenon and *fascia cruris*.

### Injury risk analysis

An in silico tendon loading experiment was performed to assess whether changes in free tendon length influence the injury risk profile. The AT in the FE models was sequentially loaded, in 100 N increments, from 2500 N to 3500 N. Assuming ATs tend to rupture when subjected to strains above 10% [58], values of maximum principal strain (MPS) in the AT were evaluated to investigate its risk of injury. Using a script developed in MATLAB R2019a (Math Works), the volume of elements in the model exhibiting an MPS above 10% was recorded. Elements close to boundary conditions were excluded under Saint–Venant's principle [17]. Like in previous studies [44, 47, 48], an AT rupture was considered to have occurred if a continuous group of elements, with a volume of at least 3 mm^3^, exhibited an MPS above 10%. The smallest load meeting, the rupture criterion and the respective rupture location were noted for each model.

### Sensitivity analyses on subtendon division and interaction

Sensitivity analyses were conducted to assess the influence of geometrical variations in subtendon division and subtendon interaction in the experiment results.

**Figure 2:**
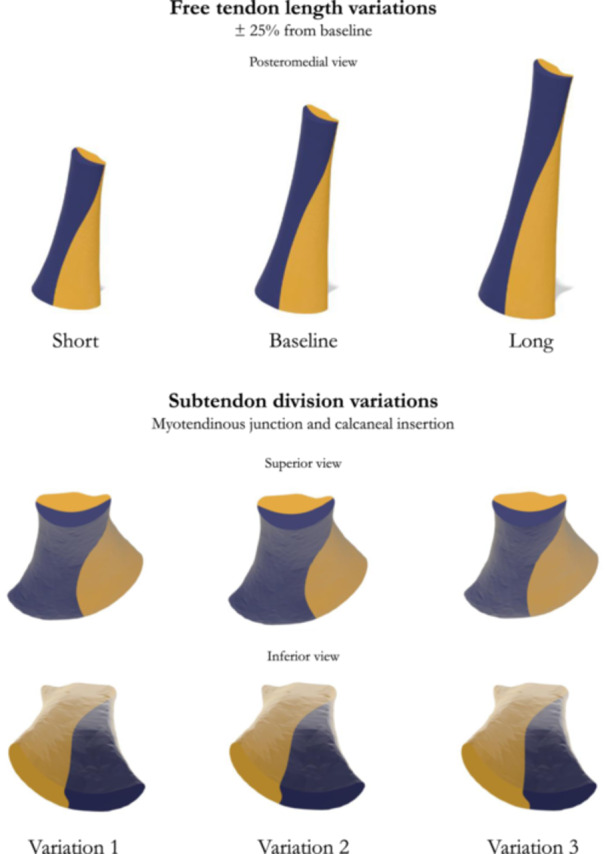
Models included in the study with length and subtendon twist variations.

To create these additional variations, a lattice with eight vertices distributed across two levels was created in Blender, with the upper limit on the distal soleus MTJ and aligned with the models' longitudinal and transversal axes. The four lower points were rotated in the same plane about a pivot point calculated from the centroid of these four points. Each point was rotated ±22.5 degrees, effectively creating two additional models of subtendon division comprising small variations in subtendon twisting (Figure 2) for the *short*, baseline and *long* free tendon models.

Because the FFD method used throughout this study could have unintentionally introduced changes in the cross‐sectional area (CSA) near the MTJ, this feature was evaluated in all models by conducting additional analyses. Differences in CSA were adjusted using FFD in the *short* and *long* models, similar to what was described for variations in free tendon length. Briefly, the square made from selecting the four lattice points at the MTJ was scaled up or down to match the CSA in the baseline model at the MTJ. General geometric features of the AT in the free tendon length and subtendon division variation models can be found in Table 2.

**Table 2:** Geometric features of the models.

Model	ST division	FT Length (mm)	Volume (mm^3^)	CSA at MTJ (mm^2^)	CSA at insertion (mm^2^)	
					Soleus ST	Gastrocnemius ST
Short	Variation 1	46.6	12527	57.3	44.57	42.09
	Variation 2				40.89	45.77
	Variation 3				48.92	37.75
Baseline	Variation 1	61.9	12564	57.6	39.06	38.62
	Variation 2				35.52	42.24
	Variation 3				42.92	34.80
Long	Variation 1	77.1	12562	58.8	35.87	37.02
	Variation 2				32.43	37.02
	Variation 3				39.75	33.14

Abbreviations: CSA, cross‐sectional area; FT, Free tendon; MTJ, myotendinous junction; ST, subtendon.

Finally, the baseline model loading experiment was repeated with a no‐sliding contact condition, that is, considering the subtendons bonded, to assess the influence of decreased subtendon sliding.

### Statistical analysis

Statistical analyses were performed using Microsoft Excel for Mac V16.45 (Microsoft Corporation). Descriptive statistics are represented as means and standard deviations, except where otherwise specified. The Pearson's correlation coefficient was used to explore potential correlations between variables. Statistical significance was set at *p* < 0.01.

## RESULTS

### Injury risk analysis

**Figure 3:**
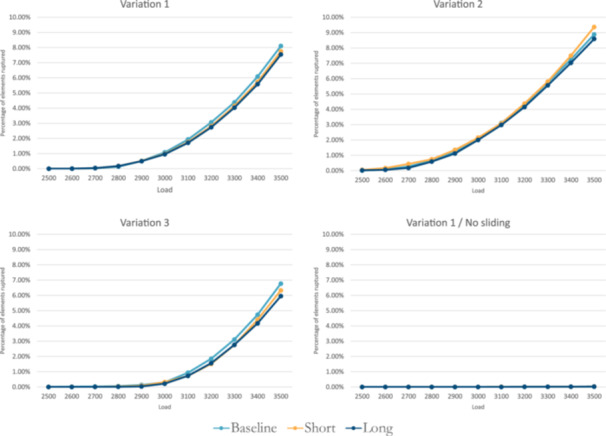
Plots of the percentage of elements exhibiting a maximum principal strain above 10% across the different loads. The model was sequentially loaded in 100 N increments, starting with 2500 N to 3500 N. N: Newtons.

**Figure 4:**
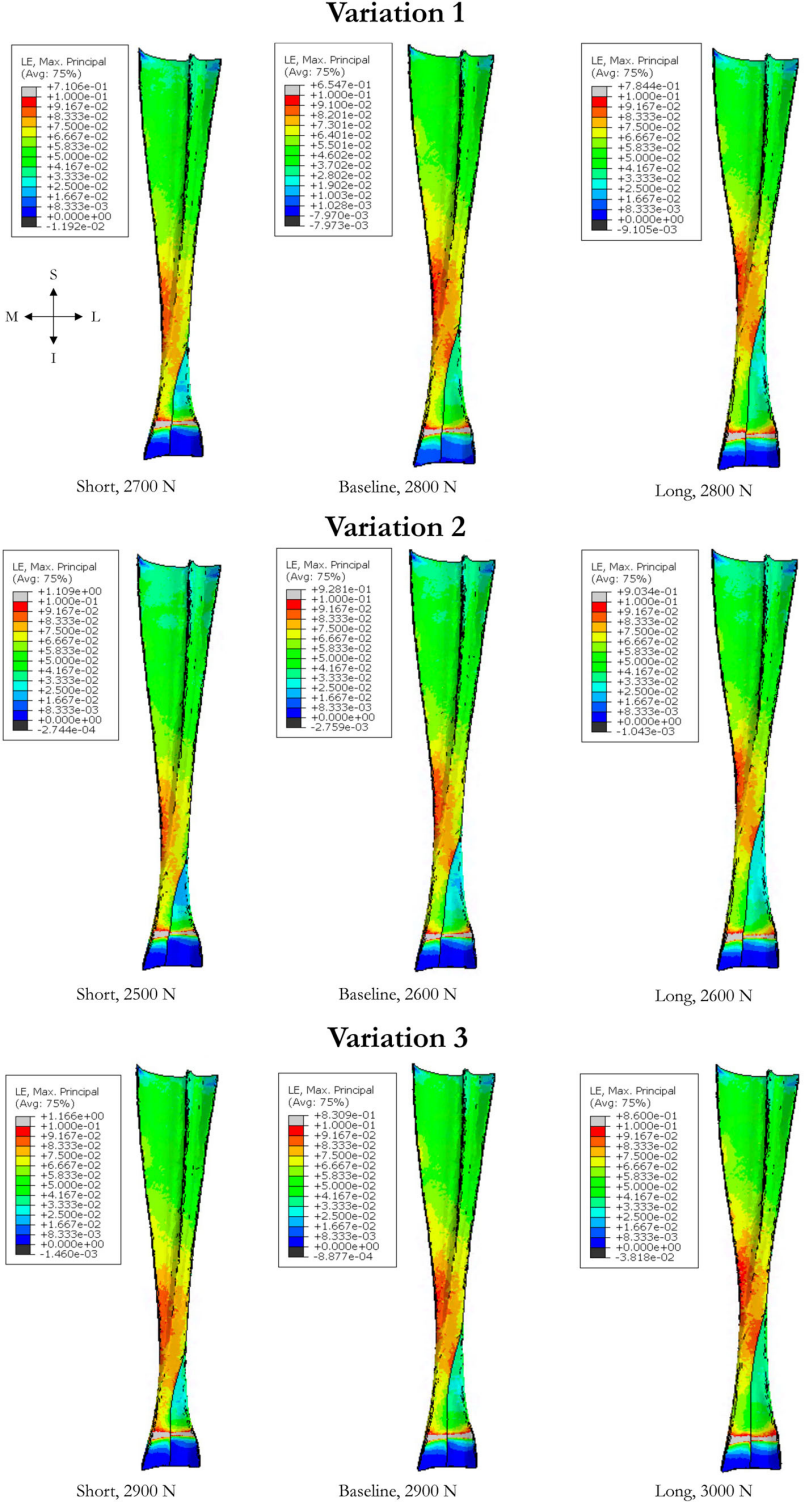
Strain distribution in the FE models at rupture load. A rupture was considered to have occurred if a continuous group of elements with a volume of at least 3 mm^3^ exhibited a maximum principal strain above 10%. N, Newtons; I, inferior; L, lateral; M, medial; S, superior.

Rupture loads were similar for *short*, baseline and *long* free tendon models (Table 3). Plots of elements exhibiting MPSs above 10% are shown in Figure 3 for the developed FE models. The rupture location was near the MTJ (Figure 4) on the medial side for all the models. This location coincided with the lowest CSA of the AT (Figure 4). The soleus subtendon exhibited the largest volume of elements, exceeding an MPS of 10%.

**Table 3:** Rupture loads in the different models. Loads are expressed in Newtons (N).

	Short	Baseline	Long
Variation 1	2700 (2700)	2800	2800
Variation 2	2500 (2600)	2600	2600
Variation 3	2900	2900	3000 (2900)

*Note*: Values between parenthesis correspond to rupture loads after correcting differences in cross‐sectional area at the myotendinous junction between models with the same subtendon division variation.

### Sensitivity analyses

Variations in subtendon division led to noticeable differences in rupture load. The percentage of the AT CSAs pertaining to the soleus and the gastrocnemius subtendons were directly (*r* = 0.87, 99% confidence interval (CI) [0.27, 0.98]) and inversely (*r* = −0.94, 99% CI [−0.99, −0.6]) correlated with the rupture load, respectively.

The small differences in the rupture load among models within the same subtendon division were not found after correcting differences in CSA at the MTJ (Table 3).

When the tendon loading experiment simulations were run with a no‐sliding condition, none of the AT models met the defined rupture criterion within the 2500–3500 N loading range. Furthermore, the number of elements evidencing MPSs above 10% was low (Figure 3).

## DISCUSSION

This study's objective was to evaluate whether variations in free tendon length influence AT's risk of injury, specifically considering the strain exhibited by the FE models during a simulated isometric ankle plantarflexion. Our investigation was motivated by previous research indicating that AT geometry, including free tendon length, might significantly affect stress distribution and, consequently, the injury risk. Using a previously validated three‐dimensional FE model incorporating both the aponeurotic and free portions of the AT, we systematically varied the free tendon length to assess its impact on strain patterns and rupture loads. Contrary to our hypothesis, the results did not demonstrate a significant influence of free tendon length on the injury risk of the AT. These findings suggest that variations in free tendon length, within the range studied, do not independently alter the mechanical behaviour of the tendon in a way that increases injury risk. Additionally, our results underscore the importance of considering other anatomical and biomechanical factors, such as subtendon CSA and sliding mechanics, which were found to be more critical determinants of tendon strain and rupture risk.

While comparing the magnetic resonance imaging (MRIs) studies of 72 patients with clinically suspected and MRI‐confirmed Achilles tendinopathy with the MRIs of 72 control subjects, Szaro et al. [16] found that the free AT length was significantly longer in the tendinopathy group than in the control group (59.7 mm vs. 38.5 mm, respectively). Conversely, other studies found no relationship between free AT length and sports activity [31] or tendinopathy [39]. The results of the present study suggest that AT length variations have little influence on the injury risk, supporting the latter studies. Tendinopathic tendons exhibit inferior material properties [42], which have been correlated with a compensatory increase in AT CSA [12, 49]. Coincidentally, in another study, Szaro et al. found that AT length and thickness measured in MRI were positively correlated [55]. Thus, it can be speculated that increased free AT length may also correlate with inferior mechanical properties. Still, more research is needed to ascertain whether increased free AT length is a consequence of altered material properties.

In the present study, variations in subtendon CSA were found to influence the risk of AT injury: rupture loads were correlated with subtendon CSAs. Previous studies have noted the significant sensitivity of AT FE models to tendon CSA [48, 49], but the interplay between soleus and gastrocnemius subtendons' CSAs is a previously unreported finding. Effectively, a reduction of the soleus subtendon CSA led to a decrease in the load needed to rupture in the simulated rupture experiment, which is related to the soleus muscle being responsible for exerting 62% of the triceps surae load in the present model [3]. Note that when most of the load had been considered to be exerted by the gastrocnemius muscles, the opposite effect would have been found.

The location of AT ruptures in the tendon midportion has been hypothesized to be related to impaired regional vascularization [7]. However, this notion of blood flow impairment in the AT midportion has been challenged by Astroöm et al. [4]. These authors used laser Doppler flow analysis to evaluate blood flow in the tendon in real‐time in 28 healthy volunteers. They found blood flow evenly distributed throughout the tendon with a slight decrease at the calcaneal insertion. In the present study, the highest AT strains were located in the section with the smallest CSA. Thus, it can be speculated that factors other than vascularization, such as a smaller CSA, may be related to degenerative changes and ruptures in the midportion.

When running the simulations with a no‐sliding condition between subtendons, the volume of total elements experiencing strains above 10% was significantly reduced. Moreover, none of the models failed in the simulated rupture experiment. One possible explanation for this finding is that by tying the subtendons, the CSA available for force transmission is considerably increased. Interestingly, experimental studies involving older subjects, both humans [17, 52] and equines [57], have reported decreased subtendon sliding, leading their authors to hypothesize that this decreased differential displacement could be a risk factor for tendinopathy [50], as this disorder is more common in older subjects. However, from this study's perspective, it could also be argued that decreased subtendon sliding may be a protective adaptation in degenerated tendons. However, further studies are needed to evaluate the role of reduced subtendon sliding in the biomechanical behaviour of the AT.

### Clinical significance

This study's results show that a decreased subtendon CSA may be a risk factor for rupture, most notably when the corresponding triceps surae muscle is the dominant force in ankle plantarflexion. Accordingly, triceps surae muscles' activation dynamics may lead to nonuniform displacement patterns in the AT, as demonstrated in an in vivo experiment by Clark and Franz [9]. In addition, previous research using biplanar ultrasound imaging has suggested that the largest displacements in the AT are located in tissue most likely belonging to the soleus subtendon [52].

The typical injury mechanism of ATs involves a forced ankle plantarflexion contraction with the knee in extension or early flexion [35]. Interestingly, concurrent activation of knee extensor muscles during plantarflexion leads to selective soleus muscle activation and decreased gastrocnemius muscle activity [53]. In addition, reduced activation of the gastrocnemius muscles has also been noted in older individuals [28]. Of note, the percentage of contribution of the soleus subtendon to the total Achilles volume ranges from 34% to 52% [41]. Thus, it can be hypothesized that decreased concurrent activation of the gastrocnemius and soleus muscles during the push‐off movement can put the tendon at risk of rupture by over‐soliciting the soleus subtendon in susceptible individuals, that is, those with a soleus subtendon CSA on the lower end range.

The ability to identify AT subtendons' distribution is currently limited [54], but as imaging methods improve, it may become a reality. Regardless, the implementation of exercises to enhance co‐contraction of gastrocnemius and soleus muscles during push‐off or changes of direction may be a valuable prevention strategy in susceptible‐to‐injury groups, for example, older athletes in sports such as basketball and soccer [14, 35] and those with previous AT tendinopathy [59].

### Limitations

This study's main limitation is the possibility of unwittingly adding unwarranted features or anatomic variations to the baseline FE model while performing FFD to model variations in free tendon length or tendinopathy. To account for this possibility, all AT models were carefully inspected, and if these unexpected concurrent variations were found, appropriate sensitivity analyses were conducted to assess their possible influence on the results obtained. This study is also based on the AT geometry of a single subject. Therefore, it could be argued that specific AT geometries and subtendon divisions may be more susceptible to AT ruptures. Two additional subtendon twist variation models were created to account for this possibility. Still, despite this, other geometrical features related to the overall shape of the tendon could influence the study's results. Finally, the model herein presented only simulates an isometric plantarflexion of the ankle, whereas considering tendon movements and tensile displacement would be a potential direction for future research.

### Future research

Assessing the specific contribution of each subtendon to the total AT volume may help detect susceptibility to injury for conditions of increased differential displacement of the subtendons. Thus, methods to assess fibre direction, dispersion or subtendon division may be of interest for biomechanical investigations and possibly the development of injury susceptibility models. Future FE studies may evaluate the gastrocnemius‐soleus subtendon dynamics, particularly in modelling the subtendon interface when simulating specific patients' situations or performing given tasks.

## CONCLUSION

Free AT length did not influence tendon strain and rupture load, provided other parameters and features were kept constant. Rupture load was sensitive to changes in subtendon CSA size. Increased force transmission between subtendons due to a decreased subtendon sliding condition caused fewer elements to experience strains above the failure criterion.

## AUTHOR CONTRIBUTIONS

Pedro Diniz conceptualized the study, created the 3D models, prepared the finite element models, collected the output data, performed the analysis of the results and drafted the manuscript. Carlos Quental conceptualized the study, prepared the finite element models, wrote the code to assign material orientations, forces exerted on the aponeurotic tendon and volume calculations and revised the manuscript. André Soares Ferreira assisted with manuscript preparation. Hélder Pereira, Frederico Ferreira and Gino M. M. J. Kerkhoffs revised the manuscript. João Folgado conceptualized the study, prepared the finite element models and revised the manuscript. All authors have read and approved the final submitted manuscript.

## CONFLICT OF INTEREST STATEMENT

The authors declare no conflicts of interest.

## ETHICS STATEMENT

Not applicable.

## Data Availability

Data is available from the corresponding author on reasonable request.

## References

[1] Ackermann, P.W. & Hart, D.A. (2016) General Overview and Summary of Concepts Regarding Tendon Disease Topics Addressed Related to Metabolic Disorders. In: Ackermann, P.W. & Hart, D.A. (Eds.) Metab influ risk tendon disord. Cham: Springer International Publishing. pp. 293–298. 10.1007/978-3-319-33943-6_28 27535271

[2] Ackermann, P.W. , Phisitkul, P. & Pearce, C.J. (2018) Achilles tendinopathy – pathophysiology: state of the art. Journal of ISAKOS, 3, 304–314. Available from: 10.1136/jisakos-2017-000164

[3] Albracht, K. , Arampatzis, A. & Baltzopoulos, V. (2008) Assessment of muscle volume and physiological cross‐sectional area of the human triceps surae muscle in vivo. Journal of Biomechanics, 41, 2211–2218. Available from: 10.1016/j.jbiomech.2008.04.020 18555257

[4] Åstroöm, M. & Westlin, N. (1994) Blood flow in the human achilles tendon assessed by laser doppler flowmetry. Journal of Orthopaedic Research, 12, 246–252. Available from: 10.1002/jor.1100120214 8164098

[5] Bandholm, T. , Sonne‐Holm, S. , Thomsen, C. , Bencke, J. , Pedersen, S.A. & Jensen, B.R. (2007) Calf muscle volume estimates: implications for Botulinum toxin treatment? Pediatric Neurology, 37, 263–269. Available from: 10.1016/j.pediatrneurol.2007.05.019 17903670

[6] Becker, J. , James, S. , Wayner, R. , Osternig, L. & Chou, L.‐S. (2017) Biomechanical factors associated with Achilles tendinopathy and medial tibial stress syndrome in runners. The American Journal of Sports Medicine, 45, 2614–2621. Available from: 10.1177/0363546517708193 28581815

[7] Chen, T.M. , Rozen, W.M. , Pan, W. , Ashton, M.W. , Richardson, M.D. & Taylor, G.I. (2009) The arterial anatomy of the Achilles tendon: anatomical study and clinical implications. Clinical Anatomy, 22, 377–385. Available from: 10.1002/ca.20758 19173244

[8] Choi, H.F. & Blemker, S.S. (2013) Skeletal muscle fascicle arrangements can be reconstructed using a Laplacian vector field simulation. PLoS One, 8, e77576. 10.1371/journal.pone.0077576 24204878 PMC3808403

[9] Clark, W.H. & Franz, J.R. (2018) Do triceps surae muscle dynamics govern non‐uniform Achilles tendon deformations? PeerJ, 6, e5182. Available from: 10.7717/peerj.5182 30013844 PMC6046199

[10] Community, B.O. (2020) Blender Foundation, Blender ‐ a 3D modelling and rendering package. Amsterdam: Stichting Blender Foundation. Retrieved from http://www.blender.org

[11] Cook, J.L. & Purdam, C.R. (2009) Is tendon pathology a continuum? A pathology model to explain the clinical presentation of load‐induced tendinopathy. British Journal of Sports Medicine, 43, 409–416. Available from: 10.1136/bjsm.2008.051193 18812414

[12] Corrigan, P. , Cortes, D.H. , Pohlig, R.T. & Grävare Silbernagel, K. (2020) Tendon morphology and mechanical properties are associated with the recovery of symptoms and function in patients with Achilles tendinopathy. Orthopaedic Journal of Sports Medicine, 8, 232596712091727. Available from: 10.1177/2325967120917271 PMC7218994 32426410

[13] van Dijk, C.N. , van Sterkenburg, M.N. , Wiegerinck, J.I. , Karlsson, J. & Maffulli, N. (2011) Terminology for Achilles tendon related disorders. Knee Surgery, Sports Traumatology, Arthroscopy, 19, 835–841. Available from: 10.1007/s00167-010-1374-z PMC3076576 21222102

[14] Diniz, P. , Abreu, M. , Lacerda, D. , Martins, A. , Pereira, H. , Ferreira, F.C. et al. (2022) Pre‐injury performance is most important for predicting the level of match participation after Achilles tendon ruptures in elite soccer players: a study using a machine learning classifier. Knee Surgery, Sports Traumatology, Arthroscopy, 30, 4225–4237. Available from: 10.1007/s00167-022-07082-4 PMC9360634 35941323

[15] Diniz, P. , Quental, C. , Violindo, P. , Veiga Gomes, J. , Pereira, H. , Kerkhoffs, G.M.M.J. et al. (2023) Design and validation of a finite element model of the aponeurotic and free Achilles tendon. Journal of Orthopaedic Research, 41(3), 534–545. Available from: 10.1002/jor.25408 35780388

[16] Drakonaki, E.E. , Gataa, K.G. & Szaro, P. (2021) The anatomical variant of high soleus muscle may predispose to tendinopathy: a preliminary MR study. Surgical and Radiologic Anatomy, 43, 1681–1689. Available from: 10.1007/s00276-021-02768-9 34032901 PMC8455493

[17] Fung, Y.C. & Tong, P. (2001) Classical and Computational Solid Mechanics. World Scientific. Available from: https://www.worldscientific.com/worldscibooks/10.1142/4134#t=aboutBook

[18] Ganestam, A. , Kallemose, T. , Troelsen, A. & Barfod, K.W. (2016) Increasing incidence of acute Achilles tendon rupture and a noticeable decline in surgical treatment from 1994 to 2013. A nationwide registry study of 33,160 patients. Knee Surgery, Sports Traumatology, Arthroscopy, 24, 3730–3737. Available from: 10.1007/s00167-015-3544-5 25697284

[19] Gasser, T.C. , Ogden, R.W. & Holzapfel, G.A. (2006) Hyperelastic modelling of arterial layers with distributed collagen fibre orientations. Journal of the Royal Society Interface, 3, 15–35. Available from: 10.1098/rsif.2005.0073 16849214 PMC1618483

[20] Handsfield, G.G. , Inouye, J.M. , Slane, L.C. , Thelen, D.G. , Miller, G.W. & Blemker, S.S. (2017) A 3D model of the Achilles tendon to determine the mechanisms underlying nonuniform tendon displacements. Journal of Biomechanics, 51, 17–25. Available from: 10.1016/j.jbiomech.2016.11.062 27919416 PMC6383718

[21] Hansen, W. , Shim, V.B. , Obst, S. , Lloyd, D.G. , Newsham‐West, R. & Barrett, R.S. (2017) Achilles tendon stress is more sensitive to subject‐specific geometry than subject‐specific material properties: A finite element analysis. Journal of Biomechanics, 56, 26–31. Available from: 10.1016/j.jbiomech.2017.02.031 28359571

[22] Järvinen, T.A.H. , Kannus, P. , Paavola, M. , Järvinen, T.L.N. , Józsa, L. & Järvinen, M. (2001) Achilles tendon injuries. Current Opinion in Rheumatology, 13, 150–155. Available from: 10.1097/00002281-200103000-00009 11224740

[23] Järvinen, T.A.H. , Kannus, P. , Maffulli, N. & Khan, K.M. (2005) Achilles tendon disorders: etiology and epidemiology. Foot and Ankle Clinics, 10, 255–266. Available from: 10.1016/j.fcl.2005.01.013 15922917

[24] de Jonge, S. , van den Berg, C. , de Vos, R.J. , van der Heide, H.J.L. , Weir, A. , Verhaar, J.A.N. et al. (2011) Incidence of midportion Achilles tendinopathy in the general population. British Journal of Sports Medicine, 45, 1026–1028. Available from: 10.1136/bjsports-2011-090342 21926076

[25] Kadlowec, J.A. , Lake, S.P. , Miller, K.S. , Soslowsky, L.J. & Elliott, D.M. (2009) A Hyperelastic Model With Distributed Fibers to Describe Human Supraspinatus Tendon Tensile Mechanics. ASME 2009 Summer Bioeng Conf Parts B American Society of Mechanical Engineers, Lake Tahoe, California, USA, 679–680

[26] Kannus, P. (1997) Etiology and pathophysiology of chronic tendon disorders in sports. Scandinavian Journal of Medicine & Science in Sports, 7, 78–85. Available from: 10.1111/j.1600-0838.1997.tb00123.x 9211608

[27] Kannus, P. & Józsa, L. (1991) Histopathological changes preceding spontaneous rupture of a tendon: a controlled study of 891 patients. The Journal of Bone & Joint Surgery, 73, 1507–1525. Available from: 10.2106/00004623-199173100-00009 1748700

[28] Kim, H. & Franz, J.R. (2021) Age‐related differences in calf muscle recruitment strategies in the time‐frequency domain during walking as a function of task demand. Journal of Applied Physiology, 131, 1348–1360. Available from: 10.1152/japplphysiol.00262.2021 34473576 PMC8560391

[29] Kinugasa, R. , Kawakami, Y. & Fukunaga, T. (2005) Muscle activation and its distribution within human triceps surae muscles. Journal of Applied Physiology, 99, 1149–1156. Available from: 10.1152/japplphysiol.01160.2004 15890750

[30] Knobloch, K. (2016) In: Ackermann, P.W. & Hart, D.A. (Eds.) Drug‐induced tendon disorders. Cham: Metab Influ Risk Tendon Disord Springer International Publishing. pp. 229–238.

[31] Kongsgaard, M. , Aagaard, P. , Kjaer, M. & Magnusson, S.P. (2005) Structural Achilles tendon properties in athletes subjected to different exercise modes and in Achilles tendon rupture patients. Journal of Applied Physiology, 99, 1965–1971. Available from: 10.1152/japplphysiol.00384.2005 16081623

[32] Lagas, I.F. , Fokkema, T. , Verhaar, J.A.N. , Bierma‐Zeinstra, S.M.A. , van Middelkoop, M. & de Vos, R.‐J. (2020) Incidence of Achilles tendinopathy and associated risk factors in recreational runners: a large prospective cohort study. Journal of Science and Medicine in Sport, 23, 448–452. Available from: 10.1016/j.jsams.2019.12.013 31892510

[33] Lantto, I. , Heikkinen, J. , Flinkkilä, T. , Ohtonen, P. & Leppilahti, J. (2015) Epidemiology of Achilles tendon ruptures: increasing incidence over a 33‐year period. Scandinavian Journal of Medicine & Science in Sports, 25, e133–e138. Available from: 10.1111/sms.12253 24862178

[34] Lemme, N.J. , Li, N.Y. , DeFroda, S.F. , Kleiner, J. & Owens, B.D. (2018) Epidemiology of Achilles tendon ruptures in the United States: athletic and nonathletic injuries from 2012 to 2016. Orthopaedic Journal of Sports Medicine, 6, 232596711880823. Available from: 10.1177/2325967118808238 PMC6259075 30505872

[35] Lemme, N.J. , Li, N.Y. , Kleiner, J.E. , Tan, S. , DeFroda, S.F. & Owens, B.D. (2019) Epidemiology and video analysis of Achilles tendon ruptures in the National Basketball Association. The American Journal of Sports Medicine, 47, 2360–2366. Available from: 10.1177/0363546519858609 31268773

[36] Leppilahti, J. & Orava, S. (1998) Total Achilles Tendon Rupture: A Review. Sports Medicine, 25, 79–100. Available from: 10.2165/00007256-199825020-00002 9519398

[37] Maffulli, N. , Longo, U.G. , Maffulli, G.D. , Rabitti, C. , Khanna, A. & Denaro, V. (2011) Marked pathological changes proximal and distal to the site of rupture in acute Achilles tendon ruptures. Knee Surgery, Sports Traumatology, Arthroscopy, 19, 680–687. Available from: 10.1007/s00167-010-1193-2 20563556

[38] Nehrer, S. , Breitenseher, M. , Brodner, W. , Kainberger, F. , Fellinger, E.J. , Engel, A. et al. (1997) Clinical and sonographic evaluation of the risk of rupture in the Achilles tendon. Archives of Orthopaedic and Trauma Surgery, 116, 14–18. Available from: 10.1007/BF00434093 9006758

[39] Nuri, L. , Obst, S.J. , Newsham‐West, R. & Barrett, R.S. (2018) Three‐dimensional morphology and volume of the free Achilles tendon at rest and under load in people with unilateral mid‐portion Achilles tendinopathy. Experimental Physiology, 103, 358–369. Available from: 10.1113/EP086673 29205610

[40] Nyyssönen, T. , Lüthje, P. & Kröger, H. (2008) The increasing incidence and difference in sex distribution of Achilles tendon rupture in Finland in 1987–1999. Scandinavian Journal of Surgery, 97, 272–275. Available from: 10.1177/145749690809700312 18812279

[41] O'Brien, M. (2005) The anatomy of the Achilles tendon. Foot and Ankle Clinics, 10, 225–238. Available from: 10.1016/j.fcl.2005.01.011 15922915

[42] Obst, S.J. , Heales, L.J. , Schrader, B.L. , Davis, S.A. , Dodd, K.A. , Holzberger, C.J. et al. (2018) Are the mechanical or material properties of the Achilles and patellar tendons altered in tendinopathy? A systematic review with meta‐analysis. Sports Medicine, 48, 2179–2198. Available from: 10.1007/s40279-018-0956-7 29961208

[43] Park, Y.H. , Lim, J.W. , Choi, G.W. & Kim, H.J. (2019) Quantitative magnetic resonance imaging analysis of the common site of acute Achilles tendon rupture: 5 to 8 cm above the distal end of the calcaneal insertion. The American Journal of Sports Medicine, 47, 2374–2379. Available from: 10.1177/0363546519858990 31287711

[44] Quental, C. , Folgado, J. , Monteiro, J. & Sarmento, M. (2016) Full‐thickness tears of the supraspinatus tendon: a three‐dimensional finite element analysis. Journal of Biomechanics, 49, 3962–3970. Available from: 10.1016/j.jbiomech.2016.11.049 27890533

[45] Ribeiro, N. , Fernandes, P. , Lopes, D. , Folgado, J. & Fernandes, P. (2009) 3‐D Solid and Finite Element Modeling Of Biomechanical Structures ‐ A Software Pipeline. Lisbon, Portugal. Available from: http://citeseerx.ist.psu.edu/viewdoc/download?doi=10.1.1.544.2385%26rep=rep1%26type=pdf

[46] Sheth, U. , Wasserstein, D. , Jenkinson, R. , Moineddin, R. , Kreder, H. & Jaglal, S.B. (2017) The epidemiology and trends in management of acute Achilles tendon ruptures in Ontario, Canada: a population‐based study of 27 607 patients. The Bone & Joint Journal, 99–B, 78–86. Available from: 10.1302/0301-620X.99B1.BJJ-2016-0434.R1 28053261

[47] Shim, V.B. , Fernandez, J.W. , Gamage, P.B. , Regnery, C. , Smith, D.W. , Gardiner, B.S. et al. (2014) Subject‐specific finite element analysis to characterize the influence of geometry and material properties in Achilles tendon rupture. Journal of Biomechanics, 47, 3598–3604. Available from: 10.1016/j.jbiomech.2014.10.001 25458149

[48] Shim, V.B. , Handsfield, G.G. , Fernandez, J.W. , Lloyd, D.G. & Besier, T.F. (2018) Combining in silico and in vitro experiments to characterize the role of fascicle twist in the Achilles tendon. Scientific Reports, 8, 13856. Available from: 10.1038/s41598-018-31587-z 30218024 PMC6138712

[49] Shim, V.B. , Hansen, W. , Newsham‐West, R. , Nuri, L. , Obst, S. , Pizzolato, C. et al. (2019) Influence of altered geometry and material properties on tissue stress distribution under load in tendinopathic Achilles tendons: a subject‐specific finite element analysis. Journal of Biomechanics, 82, 142–148. Available from: 10.1016/j.jbiomech.2018.10.027 30424837

[50] Slane, L.C. & Thelen, D.G. (2015) Achilles tendon displacement patterns during passive stretch and eccentric loading are altered in middle‐aged adults. Medical Engineering & Physics, 37, 712–716. Available from: 10.1016/j.medengphy.2015.04.004 25962378 PMC4478106

[51] Steinmann, S. , Pfeifer, C.G. , Brochhausen, C. & Docheva, D. (2020) Spectrum of tendon pathologies: triggers, trails and end‐state. International Journal of Molecular Sciences, 21, 844. Available from: 10.3390/ijms21030844 32013018 PMC7037288

[52] Stenroth, L. , Thelen, D. & Franz, J. (2019) Biplanar ultrasound investigation of in vivo Achilles tendon displacement non‐uniformity. Translational Sports Medicine, 2, 73–81. Available from: 10.1002/tsm2.61 31008448 PMC6472705

[53] Suzuki, T. , Chino, K. & Fukashiro, S. (2014) Gastrocnemius and soleus are selectively activated when adding knee extensor activity to plantar flexion. Human Movement Science, 36, 35–45. Available from: 10.1016/j.humov.2014.04.009 24922619

[54] Szaro, P. , Cifuentes Ramirez, W. , Borkmann, S. , Bengtsson, A. , Polaczek, M. & Ciszek, B. (2020) Distribution of the subtendons in the midportion of the Achilles tendon revealed in vivo on MRI. Scientific Reports, 10, 16348. Available from: 10.1038/s41598-020-73345-0 33004938 PMC7529808

[55] Szaro, P. & Ghali Gataa, K. (2021) The correlations between dimensions of the normal tendon and tendinopathy changed Achilles tendon in routine magnetic resonance imaging. Scientific Reports, 11, 6131. Available from: 10.1038/s41598-021-85604-9 33731785 PMC7969943

[56] Tallon, C. , Maffulli, N. & Ewen, S.W.B. (2001) Ruptured Achilles tendons are significantly more degenerated than tendinopathic tendons. Medicine & Science in Sports & Exercise, 33, 1983–1990. Available from: 10.1097/00005768-200112000-00002 11740288

[57] Thorpe, C. , Udeze, C. , Birch, H. , Clegg, P. & Screen, H. (2013) Capacity for sliding between tendon fascicles decreases with ageing in injury prone equine tendons: a possible mechanism for age‐related tendinopathy? European Cells and Materials, 25, 48–60. Available from: 10.22203/eCM.v025a04 23300032

[58] Wren, T.A.L. , Yerby, S.A. , Beaupré, G.S. & Carter, D.R. (2001) Mechanical properties of the human Achilles tendon. Clinical Biomechanics, 16, 245–251. Available from: 10.1016/S0268-0033(00)00089-9 11240060

[59] Yasui, Y. , Tonogai, I. , Rosenbaum, A.J. , Shimozono, Y. , Kawano, H. & Kennedy, J.G. (2017) The risk of Achilles tendon rupture in the patients with Achilles tendinopathy: Healthcare Database Analysis in the United States. BioMed Research International, 2017, 1–4. Available from: 10.1155/2017/7021862 PMC5429922 28540301

[60] Yin, N.‐H. , Fromme, P. , McCarthy, I. & Birch, H.L. (2021) Individual variation in Achilles tendon morphology and geometry changes susceptibility to injury. eLife, 10, e63204. Available from: 10.7554/eLife.63204 33588992 PMC7886322

